# Combined head accelerometry and EEG improves the detection of respiratory‐related cortical activity during inspiratory loading in healthy participants

**DOI:** 10.14814/phy2.15383

**Published:** 2022-07-11

**Authors:** Anna L. Hudson, Nicolas Wattiez, Xavier Navarro‐Sune, Mario Chavez, Thomas Similowski

**Affiliations:** ^1^ College of Medicine and Public Health Flinders University Adelaide Australia; ^2^ Neuroscience Research Australia and University of New South Wales Sydney Australia; ^3^ Sorbonne Université INSERM UMRS1158 Neurophysiologie Respiratoire Expérimentale et Clinique Paris France; ^4^ Sorbonne Université INSERM UMR 1127, CNRS UMR 7225, Institut du Cerveau et de la Moelle Épinière Paris France; ^5^ myBrain Technologies Paris France; ^6^ AP‐HP, Groupe Hospitalier APHP‐Sorbonne Université, Hôpital Pitié‐Salpêtrière Département R3S Paris France

**Keywords:** Bereitschaftspotential, brain computer interface, classifier fusion, covariance classifier, electroencephalography

## Abstract

Mechanical ventilation is a highly utilized life‐saving tool, particularly in the current era. The use of EEG in a brain–ventilator interface (BVI) to detect respiratory discomfort (due to sub‐optimal ventilator settings) would improve treatment in mechanically ventilated patients. This concept has been realized via development of an EEG covariance‐based classifier that detects respiratory‐related cortical activity associated with respiratory discomfort. The aim of this study was to determine if head movement, detected by an accelerometer, can detect and/or improve the detection of respiratory‐related cortical activity compared to EEG alone. In 25 healthy participants, EEG and acceleration of the head were recorded during loaded and quiet breathing in the seated and lying postures. Detection of respiratory‐related cortical activity using an EEG covariance‐based classifier was improved by inclusion of data from an Accelerometer‐based classifier, i.e. classifier ‘Fusion’. In addition, ‘smoothed’ data over 50s, rather than one 5 s window of EEG/Accelerometer signals, improved detection. Waveform averages of EEG and head acceleration showed the incidence of pre‐inspiratory potentials did not differ between loaded and quiet breathing, but head movement was greater in loaded breathing. This study confirms that compared to event‐related analysis with >5 min of signal acquisition, an EEG‐based classifier is a clinically valuable tool with rapid processing, detection times, and accuracy. Data smoothing would introduce a small delay (<1 min) but improves detection results. As head acceleration improved detection compared to EEG alone, the number of EEG signals required to detect respiratory discomfort with future BVIs could be reduced if head acceleration is included.

## 1 INTRODUCTION

Resting breathing is usually controlled by the automatic respiratory centers in the pontomedullary regions. Respiratory‐related motor cortical activity provides additional descending inputs to the respiratory motoneurons to compensate for decreased automatic ventilation, to compensate for inspiratory load‐capacity imbalance, or to perform voluntary tasks. Electroencephalography activity (EEG) can be used to detect respiratory‐related cortical activity, as a pre‐motor potential or ‘*Bereitschaftspotential*’ (Macefield & Gandevia, 1991). Pre‐motor potentials have been detected before inspiration (pre‐inspiratory potentials) or before expiration (pre‐expiratory potentials) in loaded breathing or simulated ‘patient‐ventilator asynchrony’ in healthy control participants (Raux et al., 2007a, b; Hudson et al., 2016; Morawiec et al., 2015), during resting breathing in respiratory or neurological disease (Nguyen et al., 2018; Georges et al., 2016; Launois et al., 2015) and in critically ill patients who are mechanically ventilated (Raux et al., 2019). Respiratory‐related cortical activity is usually accompanied by respiratory discomfort or dyspnoea (Morawiec et al., 2015; Nguyen et al., 2018; Georges et al., 2016; Raux et al., 2019). Thus, it was proposed that EEG could be used in a brain–ventilator interface (BVI) to detect respiratory discomfort and consequently improve treatment in mechanically ventilated patients (Navarro‐Sune et al., 2017). However, as previously stated, for a BVI to be clinically valuable, rapid processing and detection times are needed whereas event‐related analysis of EEG from waveform averages of 60+ breaths requires at least 5 min of signal acquisition.

We have developed new methodologies using continuous analysis of the EEG signals to detect respiratory‐related cortical activity (Hudson et al., 2016; Navarro‐Sune et al., 2017). The covariance‐based classifier tests for altered brain activity by first defining a reference period of EEG from covariance (or connectivity) matrices (i.e., reference prototypes) and then determines when EEG matrices differ from reference prototypes. The performance of the classifier was tested by a k‐fold cross‐validation and represented by Receiver Operating Characteristic (ROC) curves. The areas under ROC curves (AUC; range 0–1) indicate the performance of the classifier, for example, an AUC of 1 indicates perfect discrimination between the altered and reference EEG, and an AUC of 0.5 indicates random discrimination. Using 9 frontal and central EEG channels, the classifier can discriminate between resting and loaded breathing (inspiratory threshold load of 23cmH_2_0) in healthy, seated participants with a mean AUC of 0.85 (Hudson et al., 2016). Using 8–14 EEG channels, for discrimination of brain activity before and after ventilator adjustments that relieved dyspnoea in mechanically ventilated patients, the median AUC was 0.89 (Raux et al., 2019). Ideally, a BVI would have near‐perfect discrimination from as few signals as possible.

Signal artifacts are bound to interfere with classifier performances with head movements being of particular concern for EEG recordings. For example, a combination of EEG and gyroscope signals (to indicate changes in head orientation, that is, angular velocity) was better than EEG or gyroscope data alone to detect artifact from head movements that typically occur during ambulatory EEG recordings (O'Regan & Marnane, 2013). The technique of combining different signal modalities for machine learning classification is termed “fusion” whereby the signals (or features) are either integrated “early” to generate one classifier or distinct classifiers are integrated “late” to generate one final decision rule that in turn classifies the data. Breathing is associated with head movements that can become very intense when respiratory activity increases, at least in children (World Health Organisation, 2013). Thus, we tested the hypothesis that an accelerometer to detect changes in head movement (i.e., linear velocity) would improve, compared to an EEG covariance‐based classifier alone, the detection of respiratory‐related cortical activity. Accelerometer data are highly informative as they can characterize both individuals' behavior (Hossain et al., 2019; Hung et al., 2013) and cardio‐respiratory activity (Hernandez et al., 2014; Röddiger et al., 2019; Ruminski et al., 2014). Given their unobtrusive design and reduced cost, they are increasingly used in wearable technology. For comparison with previous studies (see above), we determined time‐locked changes in EEG signals, that is, pre‐inspiratory potentials, but also motor potentials that typically follow pre‐inspiratory potentials. As breathing can be associated with head movements that may contaminate EEG (see Jeran et al., 2013) we assessed time‐locked changes in head movement in resting and loaded breathing.

We measured EEG and head movement in healthy participants, for two different levels of inspiratory load, with quiet (i.e., resting) breathing as a reference period. Two postures, seated and reclined (i.e., two‐thirds supine) were also studied as clinical practice guidelines recommend that mechanically ventilated patients in the intensive care unit should be semi‐supine to decrease the risk of respiratory infections and increase the patient comfort (Dodek et al., 2004).

## 2 METHODS

The studies were carried out in 25 healthy participants (10 males) with an average (±SD) age, height, and weight of 25.2 ± 3.3 years, 1.71 ± 0.1 m, and 62.5 ± 8.7 kg. All were naive to respiratory physiology experiments. Most were non‐smokers, with 5 participants reported to have smoked previously (4 pack years or less). The study procedures were approved by the *Comité de Protection des Personnes Ile‐de‐France VI*, Groupe Hospitalier Pitie‐Salpetriere, Paris, France, conformed with the Declaration of Helsinki, except for database registration (clause 35) and participants gave informed written consent prior to the study.

### 2.1 Experimental set‐up and protocol

**FIGURE 1:**
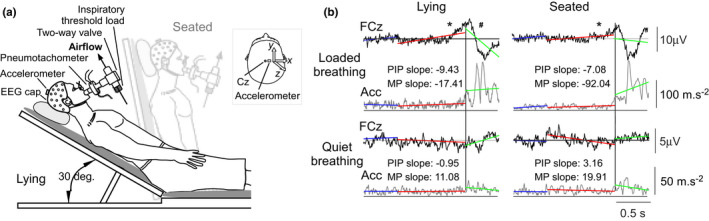
Experimental set‐up and time‐locked waveform averages of EEG and head movement. (a) Recordings were made from 32 EEG channels and a 3D accelerometer during quiet and loaded breathing in two postures. During loaded breathing, participants breathed through an inspiratory threshold load, set to either a ‘high’ (~20 cmH_2_O) or ‘low’ (~7 cmH_2_O) load. The respiratory apparatus for quiet breathing, during which participants wore nasal cannula only, is not shown. Participants were semi‐reclined (i.e. lying) or seated (light gray). The inset shows the position of the accelerometer relative to the Cz electrode. The *x*‐axis indicated right–left, the *y*‐axis posterior–anterior, and the *z*‐axis inferior–superior movements of the head. (b) Pre‐inspiratory and motor potentials at FCz (black traces) from a participant during loaded (top panels) and quiet (bottom panels) breathing in the lying and seated postures. Average waveforms of the root sum square of the accelerometer signals (Acc; gray traces) are also shown. EEG and Acc were time‐locked to the onset of inspiratory pressure (vertical line). For EEG, the slope of the signals as indicated by the red and green lines were assessed to indicate the presence of a pre‐inspiratory (*) or motor (#) potential, respectively (see Methods). To quantify head movement, the slope of the accelerometer was calculated over the same pre‐inspiratory phase (PIP) and motor phase (MP). The slope values are shown for Acc data only. This participant was breathing through a high inspiratory threshold load.

Participants were seated in a comfortable chair with neck and head support that could be reclined by 60 deg for the “lying” condition (see Figure 1). They were in a quiet, soundproof room with experimenters in an adjacent room with a one‐way mirror to monitor the participant. Electroencephalographic activity (EEG), respiratory variables, and acceleration of the head in three dimensions were recorded during four experimental conditions: Quiet breathing and loaded breathing in both the seated and lying postures.

As described previously (Hudson et al., 2016), EEG was recorded from 32 channels (ActiCap; Brain Products) according to the 10–20 system, including electrodes on both ear lobes and under the right eye to monitor electrooculographic activity. An accelerometer (3D Acceleration Sensor, Brain Products) was taped firmly to the EEG cap, close to the vertex (see Figure 1). The accelerometer weighed 8 g and had a range of ±2 g (equivalent to approx. ±19.62 m/s^2^). Each axis of the accelerometer was recorded as auxiliary channels via the EEG hardware. The component of acceleration along the *x*‐axis corresponded to right–left, the *y*‐axis to posterior–anterior, and the *z*‐axis to inferior–superior movements of the head with changes recorded as negative–positive voltages, respectively, for all axes. Online, EEG signals were referenced to FCz with a ground electrode at FPz and amplified, filtered (0.02–1000 Hz for EEG and DC‐1000 Hz for accelerometer signals), and sampled at 500 Hz.

During the quiet breathing conditions, participants wore nasal cannula attached to a differential pressure transducer (DP45‐18; Validyne) to measure nasal pressure to indicate the onset of inspiratory airflow. During the loaded conditions, participants wore a nose clip and breathed through a mouthpiece connected to a pneumotachograph (Hans Rudolph Inc) and pressure transducer (DP45‐18; Validyne) to measure airflow. Mouth pressure was also measured close to the mouth (DP15‐34; Validyne). The pneumotachograph was connected to a 2‐way valve (Hans Ruldolph Inc) so that an inspiratory load could be added to the inspiratory port but expiration was unloaded (Figure 1). End‐tidal CO_2_ was monitored at the expiratory port (Servomex). An inspiratory threshold load (Respironics) was set to be either a “high” load (~20cmH_2_O; *n* = 13 participants) or “low” load (~7 cmH_2_O; *n* = 12 participants). Respiratory variables were sampled at 2 kHz (PowerLab, ADInstruments).

Nasal pressure (quiet breathing conditions) or mouth pressure (loaded conditions) was recorded as an auxiliary channel by the EEG hardware. To time‐lock EEG and respiratory signals, a simultaneous digital trigger generated from a threshold crossing of inspiratory nasal or mouth pressure recorded on both systems. The position of the trigger was verified offline by visual inspection.

The four experimental conditions were quiet breathing in the seated and lying postures, with no instruction given in relation to breathing, and loaded breathing with an inspiratory threshold load in the seated and lying postures. Each condition lasted approximately 10 min. The quiet breathing conditions were always performed before the loading conditions to minimize participants' awareness to their breathing pattern, but the order of the postures within the quiet and loading conditions was randomized.

### 2.2 Data analysis

#### 2.2.1 Respiratory variables

For the loaded conditions, in which participants breathed through respiratory apparatus, inspiratory mouth pressure, tidal volume, inspiratory time, mean inspiratory flow, respiratory rate, and end‐tidal CO2 were measured for each breath and averaged across loaded breaths for each participant and posture. Due to technical issues, airflow and end‐tidal CO_2_ could be not measured in 9 and 2 participants, respectively, so respiratory data are missing for these participants. Ventilation during quiet breathing was not measured as a signal of nasal pressure was used to indicate the onset of inspiration only.

#### 2.2.2 Classification using machine learning techniques

EEG data, accelerometer signals, and a combination of both were tested to determine the best classification approach to discriminate between quiet and loaded breathing. Data analyses were performed using Matlab (Mathworks Inc) version 2017b. The methodology of the EEG covariance‐based classifier has been described previously (Hudson et al., 2016; Raux et al., 2019; Navarro‐Sune et al., 2017). It uses an outlier detection approach to test for “altered” brain activity compared to “reference” activity and here, was used to test between loaded and quiet breathing in both postures. The classification is based on the distance to the centroid, *d*, defined by the reference period in a Riemannian manifold. As altered breathing is represented elsewhere in the manifold, distances to the reference centroid increase (relative to the reference period) providing a single and useful metric to assess breathing‐related states by receiver operating characteristic (ROC) curves (see below).

In this paper, we introduced two improvements with respect to previous publications (Hudson et al., 2016; Raux et al., 2019; Navarro‐Sune et al., 2017)

1. a new classifier based on accelerometer data. It can be either used alone or associated with the EEG classifier (i.e., ‘Fusion’ of distances) to improve the overall performances.
2. a time buffer to average the distance of a given segment with previous distances, introducing a “smoothing” effect. Smoothed distances introduce a time delay but provide more flexibility to adapt the classifiers to the dynamics of ventilation.

Therefore, the ability of accelerometer data to discriminate between loaded and quiet breathing was tested, both independently and in combination with EEG. The effect of smoothing distances (smoothed data) was also compared to the original approach (raw data) in the different classifiers as well as the Fusion of distances method.

#### 2.2.3 EEG covariance‐based classifier

EEG from 14 frontal and central channels (F3, Fz, F4, FC5, FC1, FC2, FC6, C3, Cz, C4, CP5, CP1, CP2, CP6) were down‐sampled to 250 Hz and segmented using a 5 s sliding window with 50% overlap. To detect contaminated segments ‐mostly due to muscular and/or movement artifacts‐ we used the same technique based on *z*‐scores as Raux et al. (2019) such that segments above the 80th quantile were discarded in the analysis.

Quality segments were then band‐pass filtered (between 8 Hz and 24 Hz to enhance motor rhythms, associated with these frequencies) and used to compute EEG spatial covariance matrices. For each participant, reference activity was defined from the first 20% of EEG during quiet breathing. Then, co‐variance matrices from the remaining quiet breathing and loaded breathing recordings were tested against the reference period (see Figure 1 in Raux et al., 2019) using Riemannian distances (*d*_*E*_). The performance of the classifier to separate the loaded from quiet breathing was tested using a 10‐fold cross‐validation technique, that is, quiet breathing is divided into 10 equal parts, and 9 of these are tested against data from the loaded breathing, for 9 iterations to cover all combinations of 9 of the 10 parts of quiet breathing. This generates sensitivity and specificity data. The area under the curve (AUC) of the resultant ROC is used to indicate the performance of the classifier in each participant, with an AUC of 1 indicating perfect discrimination and AUC of 0.5 indicating random discrimination.

For clinical applications, a minimal\reduced EEG set‐up to detect altered brain activity related to breathing is preferred. Thus, all possible combinations of six electrodes only (from the 14 frontal and central electrodes, i.e., 3003 combinations) were tested to determine which set of six electrodes gave the best AUC. The AUC from the combination of electrodes F4, FC2, FC6, C3, Cz, and C4 were superior (best median AUC across all subjects) and these data only are reported for the EEG classifier.

#### 2.2.4 Accelerometer‐based classifier

Accelerometer data (simultaneous measurements in the X, Y, and Z planes, see Figure 1) were band‐pass filtered (0.1–45 Hz) and converted to root sum squared (RSS) values (i.e. RSS = √[X^2^ + Y^2^ + Z^2^]), then segmented in 50% overlapped 5‐s windows. Segments identified by the EEG z‐score artifact detection were also removed. Then, six features in frequency domain for each segment were extracted from Fast Fourier Transform (FFT): Frequency and amplitude values of the first and second dominant peaks and the mean power between 0.3 to 2 Hz and 3 to 15 Hz. The first and second dominant peaks should correspond to respiratory rate and respiratory effort (i.e., neck and jaw tremor and muscle activity; see Gresty, 1987 and below), respectively. Human head movements contain low frequencies with the maximum spectral activity expected within 0.3 and 2 Hz. The dominant peak is likely to fall within these bounds, but the average power in this band gives complementary information on the variability of the respiratory rate and non‐respiratory slow movements. Mean power between 3 and 15 Hz will capture muscle effort during both resting and loaded breathing. Scalene and sternocleidomastoid muscles in the neck which are obligatory and accessory inspiratory muscles, respectively, have bilateral synchronous activity between 4 and 20 Hz during both abrupt forward perturbations (i.e., simulating whiplash; Blouin et al., 2006) and during ramped breathing efforts (unpublished observations, Hudson et al., 2007). The average discharge rate of motor units in the human scalene muscles is 8.7 Hz during resting breathing and increases to 9.5 Hz during hypercapnic breathing which triples tidal volume (Gandevia et al., 1999). Features were organized in six‐dimensional arrays and represented in a vector space, where Euclidean distances can be used instead of Riemannian metrics. Like the EEG covariance matrices approach, the classifier used the first 20% of data during quiet breathing to generate the centroid in the vector space from which Euclidean distances are computed for every segment (*d*_*A*_).

#### 2.2.5 Distance fusion

Given that EEG and accelerometer data provide complementary information about the overall ventilatory state of participants, we tested the performance of combining the output of both systems, that is, the distance obtained by the EEG classifier, *d*_*E*_, and the distance from the accelerometer classifier, *d*_*A*_. Distance fusion was computed as follows:

1. Transform distance data to be approximately normally distributed: log(*d*_*E*_) and log(*d*_*A*_)
2. Compute *u*_*E*_ and *u*_*A*_, that is, the values corresponding to the 90th quantile of distances during the reference period (normal breathing)
3. Compute *p*_*E*_ and *p*_*A*_ with the following sigmoid function to impose values fall between 0 and 1: *p* = 1 / (1 + *e* ^*u*^ – log[*d*])
4. Obtain distance fusion *d*_*F*_ by weight averaging the transformed distances from both classifiers: *d*_*F*_ = (1−*k*) *p*_*E*_ + *k p*_*A*_

The parameter *k* was set to 0.35 after checking different weights to maximize the performance of detection measured by the AUC.

#### 2.2.6 Distance smoothing

The introduction of a time buffer allowed the application of a moving average filter to “smooth” the distances obtained for each segment by the classifiers. We used a weighted moving average filter, that is, a filter that obtains the mean value of past *N* segments and averages it with the current distance value. Given that the assessment of breathing states needs several respiratory cycles, we studied the effect of averaging *N* = 1–12 segments, which implies 5–60 s of delay to output the smoothed distance. Classifier performances, measured by the mean AUC values in all subjects, were maximal using *N* = 10 segments (50 s) in the buffer. Data that was not smoothed is referred to as “raw”.

#### 2.2.7 Time‐locked analysis of EEG: pre‐inspiratory and motor potentials

These analyses were performed using Analyzer 2.2 (Brain Products). Offline, EEG recordings were down‐sampled to 250 Hz, re‐referenced to linked earlobes, band‐pass filtered (0.5–45 Hz), and a band‐stop filter (49–51 Hz) was applied to minimize electrical noise on the signals. EEG was segmented into epochs 1500 ms before and 500 ms after the trigger at the onset of inspiratory nasal or mouth pressure for quiet or loaded breathing conditions, respectively. Artifact rejection was performed manually, based on several components (variance, value, maximum and minimum voltage, kurtosis). Epochs were then averaged to generate waveform averages for each participant and condition, with the number of epochs indicated in Table 1. FCz was analyzed for the presence of pre‐inspiratory potentials in the loaded and quiet breathing conditions (Hudson et al., 2016; Hudson et al., 2018).

**TABLE 1:** Respiratory parameters during loaded breathing

	Lying		Seated		Statistics
	High load (*n* = 13)	Low load (*n* = 12)	High load (*n* = 13)	Low load (*n* = 12)	
Mouth pressure (cmH20)	−18.12 ± 7.08	−7.94 ± 1.90	−18.68 ± 5.65	−7.58 ± 2.34	**Load *p* < 0.001** Posture *p* = 0.85 Interact. *p* = 0.40
Tidal volume^†^ (l)	0.85 ± 0.15	0.86 ± 0.24	0.90 ± 0.14	0.83 ± 0.20	Load *p* = 0.75 Posture *p* = 0.82 Interact. *p* = 0.36
Insp. time (s)	4.00 ± 1.03	3.19 ± 1.02	4.49 ± 1.87^a^	2.89 ± 0.87	**Load *p* < 0.05** Posture *p* = 0.61 **Interact. *p* < 0.05**
Mean inspiratory flow^†^ (l/s)	0.24 ± 0.09	0.31 ± 0.12	0.23 ± 0.09	0.29 ± 0.11	Load *p* = 0.21 Posture *p* = 0.42 Interact. *p* = 0.72
Ventilation^†^ (l/m)	6.63 ± 1.64	7.50 ± 1.44	6.64 ± 1.74	8.07 ± 1.90	Load *p* = 0.18 Posture *p* = 0.28 Interact. *p* = 0.29
End‐tidal CO2^‡^ (%)	2.88 ± 1.06	3.38 ± 0.85	2.62 ± 0.91	3.27 ± 0.68	Load *p* = 0.11 Posture *p* = 0.20 Interact. *p* = 0.58

*Note*: EEG and head movement were recorded from 25 participants during loaded breathing with a high or low load and quiet breathing (not shown) in two postures. The number of participants for the high and low load conditions is indicated. The statistics (2‐way repeated measures ANOVA) indicate the effect of load level, posture and their interaction on respiratory variables. Statistically significant differences are highlighted in bold typeface. ^†^ and ^**‡**^ data available for 16 and 23 participants, respectively (see Methods).

^a^ Post‐hoc difference between load level for seated posture.

A pre‐inspiratory potential was considered to be present if three criteria were met: (i) A linear relationship in the waveform average EEG with a slope greater than −0.5 μV/s was identified over 1500 ms prior to the onset of inspiration, (ii) the slope was statistically significant to zero (*F*‐test for equality of variance), and (iii) the maximal voltage of the EEG in this period did not exceed 25 μV (suggestive of artifact). A motor potential was considered to be present according to the same criteria, but with a slope greater than 5.0 μV/s over 500 ms after the onset of inspiration.

#### 2.2.8 Time‐locked analysis of head acceleration

Head acceleration signals in three dimensions were filtered as for the EEG (see above). To assess time‐locked changes in the accelerometer signals (or head movement), the RSS was calculated from 1500 ms before to 500 ms after the onset of inspiratory pressure. As for the EEG averages, the slope of the root sum square between ‐1000 ms and 0 ms (i.e., pre‐inspiratory phase) and between 0 ms and 500 ms (i.e., motor phase) was calculated for each participant and condition. For consistency, the accelerometer slope value was assigned the same sign as the EEG, that is, negative value in the up‐going direction and positive value in the down‐going direction (see Figure 1).

In addition to slope, the average amplitude of the root sum square during the pre‐inspiratory and motor phases was calculated for each participant (irrespective of load level) to quantify overall head movement in loaded and quiet breathing in both postures. Amplitude was assigned a positive value.

### 2.3 Statistics

Group data are presented as mean (SD). To compare the effect of load level (high, low) and posture (lying, seated) on the respiratory variables during the loaded conditions, a 2‐way repeated measures ANOVA was performed, with *post‐hoc* testing using the Holm‐Sidak test. A 2‐way repeated measures ANOVA was also used to compare AUC across load level and posture for each distance type with or without distance smoothing, that is, EEG classifier: *d*_*E*_ and *d*_*E*_ *’*, Accelerometer classifier: *d*_*A*_ and *d*_*A*_ *’*, Fusion distance: *d*_*F*_ and *d*_*F*_ *’*. There was no effect of load level or posture for most comparisons so data were pooled, and a 2‐way repeated measures ANOVA was then used to compare the effect of distance type (EEG, Accelerometer, and Fusion) and data extraction type (raw and smoothed) on AUC.

For pre‐inspiratory and motor potential analysis, the presence or absence of potentials was tabulated against the respiratory condition (high load, low load or quiet breathing) for each posture (lying and standing). Three‐by‐two chi‐squared tests were used to compare the incidence of potentials between conditions. In addition, two‐by‐two Fisher Exact tests were used to compare the effect of posture for each condition at a time. To compare the number of EEG epochs and accelerometer slope with different load levels and postures, a 2‐way repeated measures ANOVA was used for loaded breathing and 1‐way repeated measures ANOVA was used to compare between postures for quiet breathing. Accelerometer amplitude between loaded and quiet breathing and postures was also compared with a 2‐way repeated measures ANOVA. Exploratory *t*‐tests (i.e., uncorrected for multiple comparisons, as the accelerometer slope data were tested in the 2‐way ANOVA) were used to compare accelerometer slope in the pre‐inspiratory or motor phase for those with and without a pre‐inspiratory or motor potential, respectively.

All statistical analyses were performed with SigmaStat, version 12.0 (Systat Software Inc.).

## 3 RESULTS

### 3.1 Respiratory variables

There was no difference in ventilation between respiratory conditions (see Table 1). As expected, a greater negative mouth pressure was generated in those given an inspiratory threshold load set to a “high” setting compared to those with a “low” setting. However, there was no effect of posture on the mouth pressure generated and no interaction between posture and load. Inspiratory time was longer for loaded breathing with a high compared to low load, due to the difference in the seated posture (see Table 1), but inspiratory time was comparable between postures.

### 3.2 Classification using machine learning techniques

The ability to discriminate between loaded and quiet breathing was tested using an EEG‐ or accelerometer‐ based classifier. In addition, the combination of their output distances, that is, “Fusion”, was tested. The best combination of 6 of the 14 premotor and motor electrodes are reported (see Methods). For each classifier type (i.e., EEG or Accelerometer) there was no effect of posture or load level, as shown in Table 2. There was an effect of load for Fusion distances, with higher AUC for the high load compared to low load. Values were pooled across postures and loads to assess the effect of distance type (EEG classifier, Accelerometer classifier vs. Fusion) and data extraction type (i.e., raw versus smoothed). There was an effect of distance type (*p* < 0.001), data extraction type (*p* < 0.001) and an interaction (*p* < 0.001), with pairwise post‐hoc differences between all averages (see Table 2). The best approach to discriminate between loaded and quiet breathing was Fusion distance with smoothed data and the worst was the Accelerometer classifier with raw data. Compared to classification with EEG alone, the addition of accelerometer data, that is, Fusion, improved the AUC curve for 23/25 participants in the lying posture and 20/25 participants in the seated posture.

**TABLE 2:** Area under the curve for the performance of EEG, Accelerometer or a combination of signals to discriminate between loaded and quiet breathing

	Lying		Seated		Statistics	All
	High load (*n* = 13)	Low load (*n* = 12)	High load (*n* = 13)	Low load (*n* = 12)		Grand average (*n* = 25)
EEG *d*_*E*_	0.71 ± 0.10	0.67 ± 0.10	0.75 ± 0.10	0.69 ± 0.11	Load *p* = 0.13 Posture *p* = 0.14 Interact. *p* = 0.67	**0.71 ± 0.10**
EEG *d*_*E*_’	0.82 ± 0.11	0.81 ± 0.13	0.88 ± 0.08	0.80 ± 0.13	Load *p* = 0.22 Posture *p* = 0.36 Interact. *p* = 0.29	**0.83 ± 0.11**
Acc *d*_*A*_	0.13 ± 0.11	0.12 ± 0.04	0.14 ± 0.08	0.14 ± 0.07	Load *p* = 0.80 Posture *p* = 0.42 Interact. *p* = 0.63	**0.13 ± 0.08**
Acc *d*_*A*_’	0.58 ± 0.13	0.58 ± 0.09	0.59 ± 0.19	0.49 ± 0.16	Load *p* = 0.29 Posture *p* = 0.26 Interact. *p* = 0.13	**0.56 ± 0.15**
Fusion *d*_*F*_	0.76 ± 0.11	0.69 ± 0.10	0.80 ± 0.11	0.71 ± 0.12	**Load *p* < 0.05** Posture *p* = 0.18 Interact. *p* = 0.74	**0.74 ± 0.12**
Fusion *d*_*F*_’	0.92 ± 0.07	0.87 ± 0.09	0.93 ± 0.07	0.83 ± 0.17	**Load *p* < 0.05** Posture *p* = 0.65 Interact. *p* = 0.35	**0.89 ± 0.11**

*Note*: AUC values are given for each posture and level of load to indicate the ability of an EEG covariance‐ or Accelerometer (Acc) ‐based classifier to detect altered brain activity during loaded breathing, as assessed using the distances of these classifiers, *d*_*E*_ and *d*_*A*_, respectively. The combination of inputs, that is, Fusion of distances, *d*_*F*_, is also shown. The classifier was tested with “raw” data of one 5‐s window or “smoothed” data over 50 s with the later indicated by the comma postfix. The statistics indicate the effect of load level, posture and their interaction (interact.) on AUC. Data were pooled across postures and loads to Grand Averages as shown on the right (see also Figure 2).

### 3.3 Pre‐inspiratory and motor potentials

A longer inspiratory time in the high compared to low load condition resulted in a difference in the numbers of epochs available for EEG waveform averages from the 10‐min recording time during loaded breathing (Table 3). There were more epochs in the low load in the seated posture. As shown in Figure 3, there was no difference in the incidence of pre‐inspiratory potentials between respiratory conditions, that is high load vs low load vs quiet breathing, in the lying (*p* = 0.07, Chi^2^) or seated (*p* = 0.08, Chi^2^) postures. There was also no difference in incidence between postures in the high load (*p* = 1.0, Fisher Exact Test), low load (*p* = 1.0, Fisher Exact Test), or quiet breathing (*p* = 0.68, Fisher Exact Test) conditions (Table 3).

**TABLE 3:** Time‐locked EEG and accelerometer data during loaded and quiet breathing

	Lying		Seated			Quiet breathing (*n* = 25)	Statistics loaded breathing	Statistics quiet breathing
	High load (*n* = 13)	Low load (*n* = 12)	Quiet breathing (*n* = 25)	High load (*n* = 13)	Low load (*n* = 12)			
Average (range) EEG epochs	65 (24–127)	82 (48–127)	161 (106–212)	64 (23–134)^a^	92 (60–139)^b^	155 (70–212)	**Load *p* < 0.05** **Posture *p* < 0.05** **Interact. *p* < 0.05**	Posture *p* = 0.55
Pre‐insp. potential incidence (%)	31 (*n* = 4)	8 (*n* = 1)	16 (*n* = 4)	23 (*n* = 3)	8 (*n* = 1)	8 (*n* = 2)	Posture (high load) *p* = 1.0 Posture (low load) *p* = 1.0	Posture *p* = 0.68
Motor potential incidence (%)	23 (*n* = 3)	17 (*n* = 2)	4 (*n* = 1)	8 (*n* = 1)	8 (*n* = 1)	8 (*n* = 2)	Posture (high/low load) *p* = 0.6/1.0	Posture *p* = 1.0
Pre‐insp accel slope (ms^−2^/s)	−7.70 ± 8.20	0.17 ± 5.18	−1.17 ± 5.22	−2.97 ± 5.93	−1.22 ± 7.08	−3.84 ± 7.19	**Load *p* < 0.05** Posture *p* = 0.31 Interact. *p* = 0.07	**Posture *p* < 0.05**
Motor accel slope (ms^−2^/s)	29.22 ± 124.55	−5.08 ± 35.72	5.55 ± 15.70	−5.85 ± 90.95^c^	11.66 ± 36.13	21.33 ± 29.21	Load *p* = 0.79 Posture *p* = 0.45 **Interact. *p* < 0.05**	**Posture *p* < 0.05**

The number of artifact‐free EEG epochs from 10 min of loaded (high or low) and quiet breathing in the lying and seated postures are shown. These epochs were used for waveform averages of EEG at FCz to detect pre‐inspiratory (insp) and motor potentials. In addition, from waveform averages of the root sum square of the accelerometer signals, the accelerometer (accel) slope was calculated over 1 s prior to (pre‐insp) or 0.5 s after (motor) the onset of inspiration. See Figure 3b for the comparison of accelerometer slope in participants with or without a pre‐inspiratory potential. The statistics for loaded breathing indicate the effect of load level, posture and their interaction (interact.) or the effect of posture alone when load levels were tested independently (e.g., Fisher Exact tests for incidence). The statistics for quiet breathing indicate the effect of posture.

^a^ Post‐hoc difference between load level for seated posture.

^b^ Post‐hoc difference between postures for low load.

^c^ Post‐hoc difference between postures for high load.

The incidence of motor potentials between respiratory conditions, that is, high load versus low load versus quiet breathing, differed in the lying (*p* = 0.02, Chi^2^) but not the seated (*p* = 0.8, Chi^2^) posture (see Figure 3A). There was no difference in incidence between postures in the high load (*p* = 0.59, Fisher Exact Test), low load (*p* = 1.0, Fisher Exact Test) or quiet breathing (*p* = 1.0, Fisher Exact Test) conditions (Table 3). Few participants had both a pre‐inspiratory and motor potential and only with a high load; two participants in the lying posture and one participant in the seated posture.

### 3.4 Time‐locked head acceleration

As for EEG potentials, the accelerometer signals were time‐locked to the onset of inspiration to determine if changes in head movement between quiet and loaded breathing affect the interpretation of EEG potentials. Table 3 summarizes the effect of load level and/or posture on accelerometer slope across all participants for loaded and quiet breathing during the pre‐inspiratory and motor phases. In relation to EEG potentials, the slope was steeper for those with a pre‐inspiratory potential (*n* = 4) compared to those without (*n* = 9) for the high load in the lying posture (Figure 3B).

Comparison of the average amplitude of the accelerometer revealed that head movement was greater during loaded breathing than quiet breathing, for both the pre‐inspiratory (*p* < 0.001) and motor (*p* < 0.01) phases (Figure 4). There was no main effect of posture on pre‐inspiratory (*p* = 0.33) or motor (*p* = 0.12) head movement. Due to an interaction between condition and posture for pre‐inspiratory movement (*p* < 0.001), the effect of posture differed for loaded and quiet breathing and greater head movement in loaded breathing was due to a difference in lying (see Figure 4).

## 4 DISCUSSION

We have shown for the first time that detection of respiratory‐related cortical activity using an EEG covariance‐based classifier is improved by the inclusion of data from an Accelerometer‐based classifier. Discrimination of loaded from quiet breathing was superior when the output of both systems was combined, i.e. Fusion of distances, and data were smoothed over 50s rather than using individual 5‐s windows. This is consistent with previous findings where angular velocity of the head (via gyroscope measures) improved the performance compared to an EEG classifier alone to detect artifacts observed during walking from head movements (O'Regan & Marnane, 2013). In addition, there was equivalent detection for small and high changes in the load to breathe and for lying and seated postures which is important for the clinical applications of this technique. With limited number of epochs available for waveform averages (see below), there was no difference in the incidence of pre‐inspiratory potentials between loaded and quiet breathing, but motor potential incidence did vary in the lying posture. Time‐locked changes in head movement, as indicated by a steeper slope in the accelerometer signal, for those with or without EEG potentials differed only for the high load condition during lying. Overall head movement, as indicated by a higher average amplitude in the accelerometer signal, was greater in loaded breathing compared to quiet breathing.

### 4.1 Advances to the development of the BVI classifier

Compared to previous discrimination analysis using the same EEG covariance‐based classifier with eight or more EEG signals (AUC of 0.85 and 0.89 in healthy participants and critically‐ill patients, respectively; see Introduction), we had an AUC of 0.71 with six EEG signals in healthy participants, averaged across load levels and postures. The lower discrimination value could be due to fewer EEG signals and/or a smaller load to breathe. The inspiratory threshold load in the present study was ~13 cmH_2_O (on average) which equates to 10%–15% of maximal inspiratory muscle strength in healthy participants, compared to 23 cmH_2_O or 20%–25% of maximal inspiratory muscle strength used previously (Hudson et al., 2016). The relative inspiratory load in critically ill patients (Raux et al., 2019) was also likely to be higher than 10%–15%, as respiratory muscle dysfunction is common in mechanically ventilated patients which reduces their inspiratory capacity. A smaller load is unlikely to explain the lower AUC seen here, as there was no effect of load level on EEG classifier performance, at least up to ~20% maximal inspiratory strength in healthy participants. Even so, in the present study, discrimination with six EEG signals was improved to AUC of 0.83 when data were smoothed over 50s.

The combination of frontal and central electrodes that provided the best AUCs was F4, FC2, FC6, C3, Cz and C4, likely to reflect respiratory‐related cortical activity in the pre‐frontal, premotor, supplementary, central, and primary motor areas consistent with our previous findings (Hudson et al., 2016; Raux et al., 2019; Navarro‐Sune et al., 2017). There was a slight lateralization in the electrode combination, with 4 of the 6 electrodes on the right side of the brain, which is incongruous with the bilateral activation of the respiratory muscles. Previously, the electrodes with the greatest “ranking”, that is influence on the detection of altered brain activity associated with loading in healthy participants had a more bilateral distribution (see Figure 6 in 10). The lateralization here may just be chance as only six electrodes were used in the EEG classifier.

The classifier with an input of accelerometer only performed poorly, especially with raw data extraction, to discriminate between loaded and quiet breathing in healthy participants in lying and seated postures. Although head movements in patients with compromised respiratory function may be greater due to recruitment of accessory respiratory muscles and/or the need to compensate for increased respiratory‐related postural perturbation, this suggests the future development of a minimal/reduced setup for a BVI needs to include at least some EEG signals to detect respiratory‐related cortical activity.

The best discrimination resulted from the Fusion of distances analysis, that combined EEG and Accelerometer classifier output distances, using smoothed data, with an AUC of 0.89. The improved Fusion AUC value suggests that EEG and head acceleration provide complementary data on respiratory‐related cortical activity. It also appears to be a more sensitive method for detection of altered brain activity as the AUC were higher for the high compared to the low load for Fusion distances, whereas the AUC for the EEG and Accelerometer classifiers were comparable across load levels. Assuming the relative inspiratory load was similar or inconsequential (see above), this is comparable to discrimination in critically ill patients before and after adjustment of ventilatory settings, but here, was achieved using seven signals (6 EEG + Accelerometer) compared to 8 or 14 EEG signals in critically ill patients (Raux et al., 2019). The application of a moving average filter (i.e., smoothing) introduces a delay of ~50 s, but improves the stability of the metric, thus improving AUCs.

EEG and Accelerometer data were combined using a type of “classifier fusion” known as “score fusion”, where the outputs (i.e., distances) of the classifiers are combined with a fixed rule, that is, sum of distances. Here, the AUC was maximized when the relative weight of the EEG to Accelerometer inputs were 0.75 to 0.35, respectively. Of all the classifier fusion methods tested, O'Regan and Marnane (2013) also found that score fusion using the sum rule provided optimal results to detect head movement artifacts from a combination of EEG and gyroscope data. Although their results of score fusion (sum rule) were matched by “feature fusion”, that is, early integration of EEG and gyroscope signals to generate one classifier, the authors endorsed score fusion methodology as it has the practical advantage of being more robust to the loss of one of the signals (O'Regan & Marnane, 2013). This attribute would also be beneficial for the intended clinical application of a BVI that combines EEG and head acceleration data in the mechanically ventilated patients.

We did not measure respiratory discomfort to demonstrate a direct link between changes in brain activity and head movement (i.e., Fusion) with dyspnoea, but this has been demonstrated previously for brain activity in healthy participants and clinical populations (see Introduction). Thus, a Fusion classifier is feasible as part of a BVI to detect respiratory discomfort and improve ventilator settings. Practically, the reference condition would be one in which respiratory discomfort is minimized (self‐reported dyspnoea or assessed using the respiratory distress observation scale in non‐communicative patients), which unsurprisingly, is also associated with improved ventilatory settings, for example, increased tidal volume and ventilation (see Raux et al., 2019). Then, deviation from this baseline ‘comfort’ can be detected and an alarm raised for clinicians to review ventilator settings. This would be an individualized approach for each patient. Using brain activity only, the median AUC was 0.89 but varied between 0.36 and 1.0. It remains to be determined if addition of head movement and Fusion classification can improve the detection of altered brain activity associated with respiratory discomfort in mechanically ventilated patients. In addition, although theoretically possible (Yger et al., 2017) and piloted in our laboratory (see Hudson et al., 2016), classification of brain activity and head movement within a ‘real‐time’ domain following classifier set‐up (i.e., learning the reference period) of ~1 min needs to be demonstrated.

### 4.2 Time‐locked changes in EEG and head movement

**FIGURE 2:**
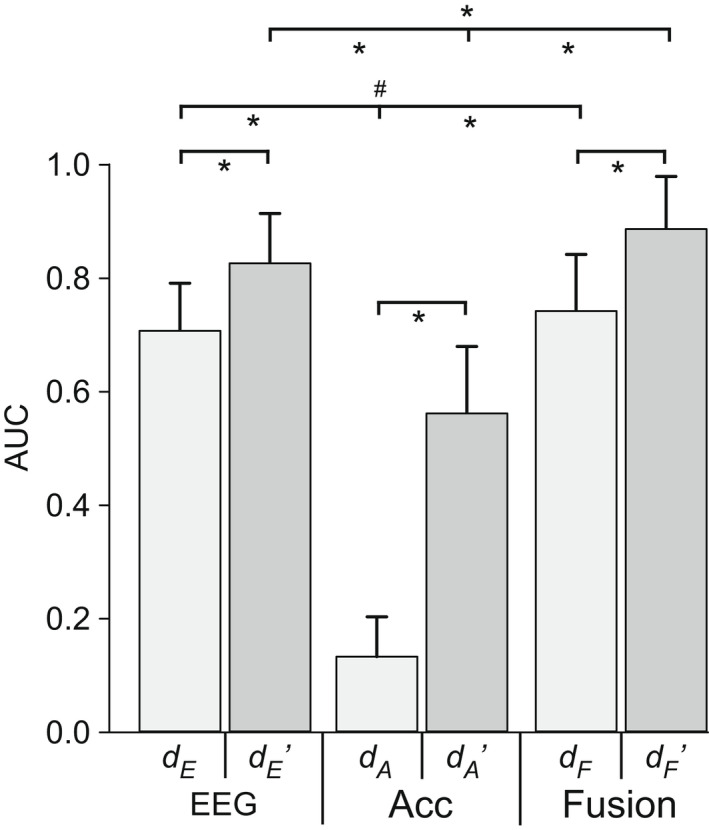
Areas under the curve for EEG‐ and Accelerometer‐ based classifiers and Fusion distances, pooled across postures and loads. Areas under the curve (AUC) for detection of loaded breathing from the reference period of quiet breathing for classifier inputs of EEG or Accelerometer (Acc) or Fusion (combination of EEG and Acc output distances) with data extraction from raw (d_E_, d_A,_ and d_F_) or smoothed data (d_E_’, d_A_’, and d_F_’; see Methods). Two‐way repeated measures ANOVA revealed an effect of analysis input, data extraction, and an interaction, such that all pairwise post‐hoc comparisons were significant, *p < 0.001, ^#^ *p* < 0.05. Mean (SD) data for 25 participants.

Previously with a similar “low” load (mean 7.5 cmH20) while seated, the incidence of pre‐inspiratory potentials was 25% (Hudson et al., 2018), comparable to 8% here in seated and lying. Previously, with a similar “high” load (median 17 cmH20) while seated, the incidence of pre‐inspiratory potentials was 67% (Tremoureux et al., 2010), compared to 23% here in seated and 31% in lying. Note even higher inspiratory threshold loads (median 20 cmH_2_0 and median 23 cmH_2_O) can result in higher incidences of pre‐inspiratory potentials of 100% (Raux et al., 2007b) and 89% (Tremoureux et al., 2010), respectively. The lower incidence in the current study for the load of ~17 cmH_2_O may be explained by the lower number of epochs available, which were ~ 65 here (as each condition was restricted to 10 min) compared to 80 epochs previously (Tremoureux et al., 2010). A greater pre‐inspiratory potential incidence during quiet breathing while supine, compared to seated posture has been previously demonstrated (Launois et al., 2019). Our participants were only reclined by 60 deg rather than supine, but the apparent influence of posture is also seen in our results both for quiet and loaded breathing (Figure 2, black versus white bars).

**FIGURE 3:**
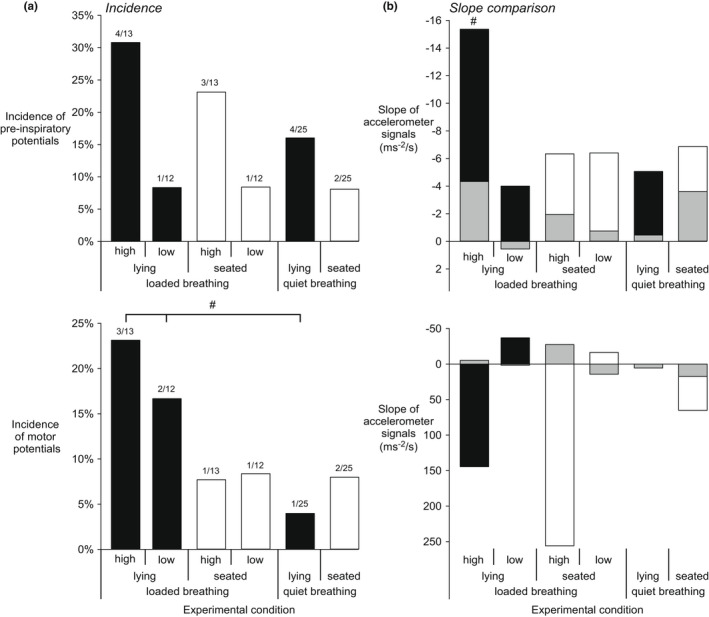
Incidence of pre‐inspiratory and motor potentials and comparison of head movement slope for participants with and without EEG potentials. (a) The incidence of pre‐inspiratory (top panel) and motor (lower panel) potentials in lying (black bars) and seated (white bars) postures for different levels of inspiratory threshold loading (high or low, see Methods) and quiet breathing. The number of participants/total participants for each condition are shown above each column. The incidence or pre‐inspiratory potentials was similar in each condition, but the incidence of motor potentials was different between conditions in the lying posture (^#^ *p* < 0.05, Chi‐squared test). (b) The slope of the root sum square of the accelerometer signals was calculated over the same pre‐inspiratory and motor phases (see Methods). The mean slope for those with a pre‐inspiratory (top panel) or motor (lower panel) potential (from panel A) are shown in black and white for the lying and seated postures, respectively. The slope for those without a pre‐inspiratory or motor potential data are shown in gray for both postures. For clarity, standard deviations are not shown but averaged 6.31 (range 4.87–9.12) ms^−2^/s when calculated for pre‐inspiratory slopes and 58 (range 12.08–245.36) ms^−2^/s for motor slopes. The slope could not be compared for conditions when only one participant had a pre‐inspiratory or motor potential as indicated in panel A (these data had a SD of zero). The slope differed between those with and without a pre‐inspiratory potential with the high load in the lying posture only (^#^ *p* < 0.05, *t*‐test). Note the change in scales for top and lower panels.

**FIGURE 4:**
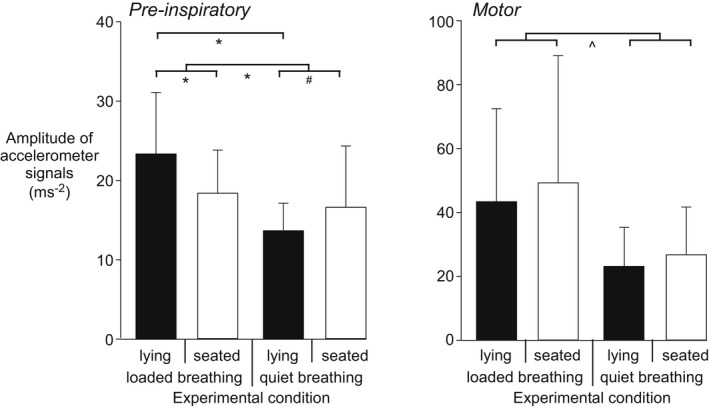
Head movement during pre‐inspiratory and motor phases of loaded and quiet breathing. Average amplitude of the root sum square of the accelerometer signals was calculated over the pre‐inspiratory (left panel) and motor (right panel) phases (see Methods). High and low load levels were pooled for loaded breathing, and a two‐way repeated measures ANOVA test revealed an effect of condition (i.e., loaded vs. quiet breathing) but no main effect of posture (lying vs. seated). Head movement was greater for loaded than quiet breathing for both the pre‐inspiratory and motor phases. However, due to an interaction between condition and posture for pre‐inspiratory movement, the effect of posture differed for loaded and quiet breathing and greater head movement in loaded breathing was due to a difference in lying. Mean (SD) data for 25 participants and all significant main and pairwise post‐hoc comparisons are shown, *p < 0.001, ^*p* < 0.01, ^#^ *p* < 0.05.

As discussed previously (Hudson et al., 2016), the pre‐inspiratory potential methodology was again limited in its ability to detect cortical‐related activity in response to smaller loads. As the AUC for discrimination between quiet and loaded breathing with a small load ranged from 0.70 to 0.88 and was not different to discrimination for high loads, it implies a limitation of the pre‐inspiratory potential technique, rather than a lack of cortical activity.

The time‐locked averages of accelerometer data reveal that over the second prior to the start of inspiratory pressure, the increases in head movement (i.e., steeper accelerometer slope) were greater when participants were breathing with a high load. The overall head movement (i.e., greater accelerometer amplitude) was also greater both prior to and after the onset of the breath in loaded compared to quiet breathing. To our knowledge, this is the first demonstration in adults that the amplitude of head movements increases when breathing becomes loaded. This information may be used to validate contactless‐respiratory monitoring in patients with acute or chronic respiratory disorders (e.g., Lee et al., 2021; Janssen et al., 2016). Given the slope was calculated from the root sum square of the signals in three dimensions, the direction of the movement is not identified, but assumed in these healthy participants to represent head extension due to bilateral recruitment of the sternocleidomastoid muscles in anticipation of having to generate a bigger negative pressure to produce inspiratory airflow (Hudson et al., 2007).

Head movement, as indicated by the slope of the signal, was bigger in those with a pre‐inspiratory potential compared to those without in the high load condition in the lying posture. We cannot compare the effect of head movement in the low load condition as only one participant had a pre‐inspiratory potential for both postures. There was no apparent effect of head movement on the detection of motor potentials, but again not all conditions could be compared due to low motor potential incidence. The influence of artefactual head movement on detection of EEG potentials in loaded breathing and in clinical populations (see Introduction for references) cannot be determined from the present study, given the low incidence of pre‐inspiratory potentials across conditions, but our data provides rationale to study this further.

## 5 CONCLUSIONS

In summary, we have demonstrated that the inclusion of an accelerometer should be considered in the further development of a BVI to detect respiratory‐related cortical activity. This should be tested in clinical populations as well as in reliability studies to confirm which EEG signals should be included with the accelerometer signal for fast and accurate detection of respiratory discomfort.

## AUTHOR CONTRIBUTIONS

Anna L. Hudson, Mario Chavez, and Thomas Similowski conceived and designed the study. Anna L. Hudson collected the data. Anna L. Hudson, Nicolas Wattiez, and Xavier Navarro‐Sune analyzed the data. All authors interpreted the data. Anna L. Hudson drafted the manuscript, and all authors critically revised the manuscript. All authors approved the final version of the manuscript submitted for publication and all persons who qualify for authorship are listed.

## CONFLICT OF INTEREST

M.Chavez and T.Similowski are listed as inventors on several patent applications and granted patents describing EEG processing methods to identify the improper settings of a mechanical ventilator. These patents are owned jointly by their employers (Sorbonne Université, Assistance Publique Hôpitaux de Paris, Inserm and CNRS, Paris, France). A pre‐licensing contract has been signed between Sorbonne Université and My Brain Technologies Ltd to develop and market a clinical device from the existing patents.

## FUNDING INFORMATION

Anna L. Hudson was funded by an Australian Academy of Science “France‐Australia Science Innovation Collaboration” Early Career Fellowship. The study was supported by Air Liquide Medical Systems and *Association pour le Développement et l'Organization de la Recherche en Pneumologie et sur le Sommeil (ADOREPS)* who also served as its legal sponsor according to French rules.
